# Exposing therapists to trauma-focused treatment in psychosis: effects on credibility, expected burden, and harm expectancies

**DOI:** 10.3402/ejpt.v7.31712

**Published:** 2016-09-06

**Authors:** David P. G. van den Berg, Berber M. van der Vleugel, Paul A.J.M. de Bont, Gwen Thijssen, Carlijn de Roos, Rianne de Kleine, Tamar Kraan, Helga Ising, Ad de Jongh, Agnes van Minnen, Mark van der Gaag

**Affiliations:** 1 Parnassia Psychiatric Institute, Den Haag, The Netherlands; 2 Community Mental Health Service GGZ Noord-Holland Noord, Alkmaar, The Netherlands; 3 Mental Health Organization (MHO) GGZ Oost Brabant Land van Cuijk en Noord Limburg, Boxmeer, The Netherlands; 4 MHO Rivierduinen, Leiden, The Netherlands; 5 Behavioural Science Institute, Radboud University Nijmegen, Nijmegen, The Netherlands; 6 Centre for Anxiety Disorders Overwaal, MHO Pro Persona, Nijmegen, The Netherlands; 7 Department of Behavioral Sciences, Academic Centre for Dentistry Amsterdam (ACTA), University of Amsterdam and VU University Amsterdam, Amsterdam, The Netherlands; 8 School of Health Sciences, Salford University, Manchester, United Kingdom; 9 Department of Clinical Psychology, VU University Amsterdam and EMGO Institute for Health and Care Research, Amsterdam, The Netherlands

**Keywords:** PTSD, prolonged exposure, EMDR, training, dissemination

## Abstract

**Background:**

Despite robust empirical support for the efficacy of trauma-focused treatments, the dissemination proves difficult, especially in relation to patients with comorbid psychosis. Many therapists endorse negative beliefs about the credibility, burden, and harm of such treatment.

**Objective:**

This feasibility study explores the impact of specialized training on therapists’ beliefs about trauma-focused treatment within a randomized controlled trial.

**Method:**

Therapist-rated (*n*=16) credibility, expected burden, and harm expectancies of trauma-focused treatment were assessed at baseline, post-theoretical training, post-technical training, post-supervised practical training, and at 2-year follow-up. Credibility and burden beliefs of therapists concerning the treatment of every specific patient in the trial were also assessed.

**Results:**

Over time, therapist-rated credibility of trauma-focused treatment showed a significant increase, whereas therapists’ expected burden and harm expectancies decreased significantly. In treating posttraumatic stress disorder (PTSD) in patients with psychotic disorders (*n*=79), pre-treatment symptom severity was not associated with therapist-rated credibility or expected burden of that specific treatment. Treatment outcome had no influence on patient-specific credibility or burden expectancies of therapists.

**Conclusions:**

These findings support the notion that specialized training, including practical training with supervision, has long-term positive effects on therapists’ credibility, burden, and harm beliefs concerning trauma-focused treatment.

**Highlights of the article:**

- Specialized training improved therapists’ credibility, burden, and harm beliefs.
- Patients’ symptom severity and treatment outcome did not affect these beliefs.
- Replication of our findings in larger cohorts with a control group is warranted.

Because there is strong empirical support for the efficacy of trauma-focused treatments such as prolonged exposure therapy (PE), eye movement desensitization and reprocessing therapy (EMDR), and cognitive therapy (Bisson, Roberts, Andrew, Cooper, & Lewis, 2013; Bradley, Greene, Russ, Dutra, & Westen, 2005), these treatments are recommended worldwide in treatment guidelines for posttraumatic stress disorder (PTSD; Forbes et al., 2010; World Health Organization, 2013). In addition, most patients with PTSD seem to have a positive attitude toward evidence-based trauma-focused treatments such as PE (Becker, Darius, & Schaumberg, 2007) and prefer this to medication (Feeny, Zoellner, Mavissakalian, & Roy-Byrne, 2009; Polusny, Erbes, & Gerould, 2014; Reger et al., 2013). PTSD is highly prevalent in patients diagnosed with a psychotic disorder (Achim et al., 2011; De Bont et al., 2015), and several trauma-focused treatments are known to be effective and safe in patients with psychosis and other severe mental illnesses (De Bont, Van Minnen, & De Jongh, 2013; Frueh et al., 2009; Mueser et al., 2015, 2008; Van den Berg et al., 2015; Van den Berg & Van der Gaag, 2012).

Nevertheless, dissemination of evidence-based trauma-focused treatments remains highly problematic (Deacon & Farrell, 2013; Foa, Gillihan, & Bryant, 2013). For example, a study using clinical data of six specialized PTSD outpatient veteran units in the USA (*n*=1,924) found that only 6.3% of the patients received at least one session of evidence-based trauma-focused treatment during the first six months of their treatment (Shiner et al., 2013). In the presence of a comorbid psychotic disorder, the situation may be even more problematic, since most therapists are reluctant to use trauma-focused treatments in patients with psychosis (Becker, Zayfert, & Anderson, 2004; Frueh, Cusack, Grubaugh, Sauvageot, & Wells, 2006; Meyer, Farrell, Kemp, Blakey, & Deacon, 2014; Salyers, Evans, Bond, & Meyer, 2004).

Together with contextual factors (e.g., insufficient time) and patient factors (e.g., poor engagement), therapist characteristics and, more specifically, therapists’ beliefs about trauma-focused treatments appear to be an important cause of underutilization of evidence-based interventions for PTSD (Becker et al., 2007; Harned, Dimeff, Woodcock, & Contreras, 2013; Meyer et al., 2014). Some therapists hold negative beliefs about the tolerability, safety, and utility of evidence-based trauma-focused treatments (Farrell, Deacon, Dixon, & Lickel, 2013; Foa et al., 2013). Based on the literature, we distinguished three types of therapist beliefs related to trauma-focused treatment that may influence therapists’ behavior in clinical practice: credibility, expected burden, and harm expectancies of trauma-focused treatment.

*Credibility* refers to therapists’ beliefs about the efficacy and utility of that particular treatment. Some therapists consider that findings on the efficacy of evidence-based treatments (mainly cognitive behavior therapy) are of little value to their clinical practice (e.g., Barlow, Levitt, & Bufka, 1999; Foa et al., 2013; Shafran et al., 2009). This is supported by a survey of 2,607 USA and Canadian psychotherapists in which significant mentors, books, training received in graduate school and informal discussions with colleagues were the most highly endorsed factors influencing clinical behavior (Cook, Schnurr, Biyanova, & Coyne, 2009). Not surprisingly, the credibility of a certain trauma-focused treatment was found to be associated with a preference for using it (Van Minnen, Hendriks, & Olff, 2010).

*Burden expectancy* concerns therapists’ beliefs that a certain treatment is burdensome for patients and therapists. Conducting trauma-focused treatments can be burdensome for both patient and therapist, albeit patients generally consider it to be tolerable, are inclined to undergo treatment again, and tend to recommend it to a friend with similar problems (Devilly & Spence, 1999). Conversely, some therapists fear that the burden associated with trauma-focused treatment may result in secondary traumatization of therapists, even though research in this field is neither clear nor consistent; however, it does not appear to be a highly prevalent problem (Elwood, Mott, Lohr, & Galovski, 2011; Van Minnen et al., 2010). Nevertheless, the expected burden for both patient and therapist may be an important factor in therapists’ reluctance to adopt trauma-focused treatments.

*Harm expectancy* refers to therapists’ (often non-empirically supported) beliefs about the possible negative consequences of using trauma-focused treatments for their patients. The most important harm expectancy of therapists is that trauma-focused treatment will destabilize the patient and exacerbate symptoms, which could result in various adverse events, for example, crises, suicide attempts, hospitalization, revictimization, and dropout (Becker et al., 2004; Foa, Zoellner, Feeny, Hembree, & Alvarez-Conrad, 2002; Gairns, Alvarez-Jimenez, Hulbert, McGorry, & Bendall, 2015; Van Minnen et al., 2010). However, the reality is that the exacerbation of PTSD symptoms in trauma-focused treatment is rare and, when it does occur, is often temporary and unrelated to treatment response (Foa et al., 2002; Jayawickreme et al., 2014; Larsen, Wiltsey Stirman, Smith, & Resick, 2015; Taylor et al., 2003). A recent review of 18 trials of PE showed that, as a result of treatment, comorbid symptoms either decline along with the PTSD symptoms or do not change at all (Van Minnen, Zoellner, Harned, & Mills, 2015). Another review showed that trauma-focused treatment does not result in large-scale dropout (Hembree et al., 2003). Moreover, in the parent trial of this study, which tested PE and EMDR in patients with psychotic disorders, exacerbation of symptoms was rare and treatment in fact resulted in a significant reduction of adversities (Van den Berg et al., 2016). Nevertheless, many therapists are still reluctant to use trauma-focused treatments due to their harm expectancies.

It is generally assumed that training reduces the negative beliefs of therapists about trauma-focused treatment and, thereby, is helpful in efforts for dissemination. Suggestions for enhancing training effects have been made, for example, by providing information that balances empirical (e.g., data supporting the rationale, effectiveness, tolerability, and safety of trauma-focused treatments) and emotional appeals (e.g., case examples) and by using exercises that prompt both explicit and implicit learning (Farrell et al., 2013). Indeed, several cross-sectional studies reported an association between previous specialized training or having more experience, and the propensity to screen for trauma/PTSD and the use of trauma-focused treatments (Becker et al., 2004; Frueh et al., 2001; Sprang, Craig, & Clark, 2008). Van Minnen et al. (2010) found specific training and experience to be positively related to therapist-rated credibility of trauma-focused treatment. Conversely, lack of training and experience were indicated as important reasons for not using trauma-focused treatments (Becker et al., 2004; Salyers et al., 2004; Van Minnen et al., 2010). Worldwide, numerous steps have been taken to improve dissemination of evidence-based trauma-focused treatments. For example, the US Department of Veterans Affairs developed programs to train therapists in the delivery of these therapies (Karlin et al., 2010). A randomized controlled dissemination trial showed that an interactive online training improved therapists’ credibility beliefs concerning exposure techniques (Harned et al., 2014). Another study found that training positively influenced beliefs concerning PE (Ruzek et al., 2016).

However, little is known about the extent to which the different phases of specialized trauma-focused treatment training influence the beliefs of therapists without previous experience in trauma-focused treatment. Therefore, to test the differential influence of these phases on therapist-rated credibility, expected burden, and harm expectancies, the present feasibility study monitored therapists’ beliefs during theoretical training, technical training, supervised practical training, and at 2-year follow-up. Also examined was whether this training resulted in sustained usage of trauma-focused treatments on the long term.

Moreover, until now, studies concerning training effects have only assessed general beliefs of therapists concerning trauma-focused treatment, independent of the specific characteristics of individual patients. However, in clinical practice, these beliefs may be influenced by patient-specific factors, such as pre-treatment severity of the patients’ symptoms or treatment outcome. Therefore, to determine whether symptom severity or treatment outcome affects therapist-rated credibility and expected burden of trauma-focused treatment, we assessed these beliefs and symptom severity at pre-treatment and post-treatment for each individual patient.

## Method

### Design

The data of this feasibility study were obtained as part of a randomized controlled trial (RCT) that found both PE and EMDR to be effective and safe in participants diagnosed with both a PTSD and a psychotic disorder (Van den Berg et al., 2015, 2016). The medical Ethics Committee of the VU University Medical Centre approved the study protocol (NL 36649.029.12). Details on the design, procedures, and instruments of this trial are available elsewhere (De Bont, Van den Berg, Van der Vleugel, et al., 2013; Van den Berg et al., 2015).

First, we describe the results of a pretest–posttest design with five repeated measurements concerning therapists’ general beliefs regarding trauma-focused treatment. Second, we test the impact of pre-treatment symptom severity on therapists’ patient-specific credibility and expected burden of trauma-focused treatment in a cross-sectional design. Then, we report the results of a pre-treatment–post-treatment analysis of the influence of treatment outcome on therapists’ beliefs concerning patient-specific credibility and expected burden of trauma-focused treatment.

### Participants

The inclusion criteria for this feasibility study were a) no previous training in PE or EMDR and b) consent to participate in monthly expert supervision sessions during the trial. The participants were 16 therapists (15 clinical psychologists and 1 psychiatrist) working at 13 mental healthcare organizations in the Netherlands. Twelve therapists were female and four were male. Their mean age was 37.1 (7.59) years, and on average, they had been working as a therapist for 8.6 (7.6) years. All therapists worked mainly with patients with psychotic disorders, were specialized in cognitive behavior therapy for psychosis, had no previous experience in trauma-focused treatment, and volunteered to participate in a trial for trauma-focused treatments in psychosis. To test the influence of symptom severity and treatment outcome on therapists’ beliefs, we included patients (with both a PTSD and a psychotic disorder; *n*=79) that received either PE or EMDR treatment during the trial from one of the 16 participating therapists.

### Measures

*Therapists’ general credibility of trauma-focused treatment* was assessed with five statements (i.e., “This treatment seems logical to me”; “This treatment seems scientific to me”; “If I had a PTSD, I would choose this treatment”; “This treatment is effective for most people”; “If a close friend or relative had PTSD, I would recommend this therapy”). Therapists responded on a visual analog scale (VAS) ranging from 0 (“disagree strongly”) to 10 (“agree strongly”) with a higher score representing a higher level of credibility of trauma-focused treatment. The therapists’ credibility assessment was inspired by the Credibility/Expectancy Questionnaire, a short measure of patient-rated credibility of treatment that has shown high internal consistency and good test–retest reliability (Devilly & Borkovec, 2000). In this study, the internal consistency of the five credibility items at the different time points ranged from 0.79≥α≤0.90.

*Therapists’ general burden and harm expectancies of trauma-focused treatment* were measured in a similar way with seven statements on a VAS ranging from 0 (“disagree strongly”) to 10 (“agree strongly”). Two statements concerned burden expectancies (i.e., “This treatment is burdensome to the patient”; “This treatment is burdensome to the therapist”) and five statements concerned harm expectancies of trauma-focused treatment (i.e., “This treatment worsens PTSD symptoms”; “This treatment worsens psychotic symptoms”; “This treatment worsens other comorbid symptoms”; “This treatment induces dropout”; “This treatment induces crisis contacts with mental healthcare or admission to hospital”). These statements were inspired by the Distress/Endorsement Validations Scale (Devilly, 2004). The internal consistency of the two burden expectancy items ranged from 0.79≥α≤0.86 at the different time points; for the five harm expectancy items, this was 0.85≥α≤0.92.

*Patient-specific credibility of trauma-focused treatment* was assessed before session 2 (the first trauma-focused treatment session) with three statements (i.e., “This is a logical treatment for this patient”; “This is an effective treatment for this patient”; “If a colleague had a similar patient, I would recommend this treatment”). Again participating therapists responded on VAS (0–10). The ratings concerned the therapist's beliefs about the treatment for that specific client.

*Patient-specific burden expectancy of trauma-focused treatment* was assessed in the same way with two statements on a VAS (i.e., “Conducting this treatment with this patient is burdensome for me”; “I feel reluctant about using this treatment with this patient”).

Independent assessors, which were successfully blinded to treatment allocation, assessed *pre-treatment symptom severity for the specific patients* (Van den Berg et al., 2015). Severity of PTSD symptoms in patients was assessed with the Clinician Administered PTSD Scale (CAPS; Blake et al., 1995). The CAPS (range 0–136) has excellent psychometric properties in terms of reliability and validity (Weathers, Keane, & Davidson, 2001). The CAPS was found to be valid and reliable in patients with severe mental illness (Mueser et al., 2001); in this study, the intra-class correlation coefficient for the CAPS for all the assessors was 0.81.

The severity of paranoid ideation in patients was measured with the Green et al. Paranoid Thought Scales (GPTS; Green et al., 2008). The GPTS is a self-report measure of paranoia and consists of 32 items concerning persecutory ideation and ideas of reference, which are scored on a Likert scale ranging from 1 (not at all) to 5 (totally). The GPTS (range 32–160) is a valid and reliable questionnaire that is sensitive to change (Green et al., 2008).

Presence of auditory verbal hallucinations (AVH) was established with the Auditory Hallucination Rating Scale (AHRS; Haddock, McCarron, Tarrier, & Faragher, 1999). We used a dichotomous outcome of the AHRS since not all patients in the present trial were actively hearing voices at that moment, which resulted in an excess of zeros in the data. The AHRS has excellent inter-rater reliability (Haddock et al., 1999).

The presence of moderate-to-high suicide risk was assessed using the suicidality section of the Mini-International Neuropsychiatric Interview-Plus (MINI-plus; Sheehan et al., 1998). The MINI-plus is a valid and reliable clinical interview (Lecrubier et al., 1997; Sheehan et al., 1997). We dichotomized the outcome of the suicidality section of the MINI-plus (no, low, moderate, or high risk) into “no or low suicide risk” and “moderate-to-high suicide risk.”

The level of social functioning of the patients was assessed with the Personal and Social Performance scale (PSP; Morosini, Magliano, Brambilla, Ugolini, & Pioli, 2000). The PSP (range 0–100, with higher scores indicating better functioning) is an assessor-rated scale to measure personal and social functioning based on the scores on several social functioning domains. The PSP is a valid and reliable test and also sensitive to change (Kawata & Revicki, 2008; Nasrallah, Morosini, & Gagnon, 2008; Patrick et al., 2009).

*Treatment outcome* was determined by subtracting the post-treatment CAPS total severity score from the pre-treatment CAPS total severity score. Both patient-specific therapist-rated credibility and burden expectancies of trauma-focused treatment were assessed at post-treatment using the same items. The internal consistencies of pre-treatment and post-treatment were 0.86≥ and ≤0.90 for credibility and 0.67≥ and ≤0.81 for burden.

### Procedures

#### Training phases

Therapists-rated credibility, expected burden, and harm expectancies of trauma-focused treatment in psychosis were assessed five times during the process of training, that is, at baseline (before the start of training), post-theoretical training, post-technical training, post-practical training, and at 2-year follow-up. Experts in the target treatments provided a 4-day training in PE (AVM and RDK) and a 4-day training in EMDR (ADJ). All therapists attended both trainings and all delivered both therapies during the trial. Standard protocols of these two guideline trauma-focused treatments were used (De Jongh & Ten Broeke, 2003; Foa, Hembree, & Rothbaum, 2007; Shapiro, 2001). Both trainings had a similar structure and took place between March and August 2011 and comprised four phases.

##### Theoretical training

The first 2 (consecutive) days of the PE and EMDR trainings were mainly theoretical, consisting of the following elements: (1) theoretical principles; (2) efficacy and safety of the treatment (also in complex patient groups); (3) treatment rationale and procedures; (4) specific techniques and skills; and (5) practicing techniques and skills in role play with peers.

###### Technical training

The third and fourth days of the two trainings were technical and were spread over a 5-month period. During this phase, the participants had to treat at least two patients with PE and two with EMDR. These sessions were videotaped, viewed, and (plenary) discussed focusing on the technical aspects of conducting the therapies.

###### Supervised practical training

Recruitment for the trial ran from September 2011 through April 2013. During this training phase, therapists treated patients and underwent monthly 4-h group supervision sessions (group size 6–8) that were led by experts (2 h by AVM in PE and 2 h by CDR or ADJ in EMDR). In these supervision sessions, video recordings of complicated treatment sessions were viewed and (plenary) discussed.

###### Two-year follow-up

At the end of the trial, the therapists resumed their regular function in clinical practice, mainly in cognitive behavior therapy for psychosis. Two years after the end of the trial, the participating therapists were surveyed about their use of PE and EMDR. The questions were “Are you still using trauma-focused treatments in patients with psychosis?”; “How many patients with a psychotic disorder did you treat with trauma-focused treatment since the closure of the trial?”; “In case you did not treat any patients with trauma-focused treatment, why not?”; and “What factor influenced your daily practice most (choices: theoretical training, technical training, supervised practical training, otherwise)?”

### Symptom severity and treatment outcome

During the supervised practical training phase, the therapists treated patients included in the trial. In this study, the 79 participating patients were randomly assigned to receive eight weekly 90-min sessions of either PE (*n*=39) or EMDR (*n*=40). All sessions were videorecorded. Session one comprised psycho-education (concerning PTSD and the rationale for treatment) and the development of a hierarchy of the worst (and most re-experienced) trauma memories. No trauma-focused treatment was provided in this first session. Therapists rated their credibility and expected burden of the treatment of every specific patient at pre-treatment (after session 1) and at post-treatment (after the last therapy session). For each patient, the independent assessors assessed pre-treatment symptom severity and post-treatment severity of PTSD symptoms.

### Analyses

Statistical analyses were conducted with SPSS 22 (IBM SPSS). In this study, we pooled the data on therapists’ beliefs of the trauma-focused treatments PE and EMDR because (1) the PE and EMDR trainings took place in the same period; (2) all therapists provided both treatments; (3) there was no difference in treatment allegiance of therapists to PE or EMDR (*t*_18_=0.000, *p*=0.999); and(4) there were no differences in efficacy (Van den Berg et al., 2015).

*Influence of training on therapist-rated credibility, burden, and harm of trauma-focused treatment*. Linear mixed models were performed to test if therapist-rated credibility, expected burden, and harm expectancies of trauma-focused treatment changed over time. Dummy variables (recodes of the time points) were used to investigate effects between the different time points, that is, during theoretical training, technical training, practical training, and the follow-up period.

*Influence of symptom severity on patient-specific credibility and burden of trauma-focused treatment*. We computed bivariate Pearson product–moment coefficients between the dependent therapist-rated variables “pre-treatment credibility of treatment” and “pre-treatment expected burden of treatment” and five independent variables representing pre-treatment symptom severity (severity of PTSD, severity of paranoid ideation, presence of AVH, presence of moderate-to-high suicide risk, and level of social functioning). Since these data are nested within therapists, we used linear mixed models with a correction for therapist level (i.e., random intercept and a random slope if that improved the model) to test these associations. The independent variables were entered (forced simultaneous entry) into two separate linear mixed models analyses (one for credibility and one for expected burden) to preserve degrees of freedom (Babyak, 2004).

*Influence of treatment outcome on patient-specific credibility and burden of trauma-focused treatment*. Paired-samples *t*-tests (completers) were used to analyze changes in the severity of PTSD symptoms and in therapist-rated credibility and burden of trauma-focused treatment, between pre-treatment and post-treatment. Then, bivariate Pearson product–moment coefficients were computed between change scores of PTSD and change in therapist-rated credibility and burden. We performed linear mixed models (intention-to-treat) with a correction for therapist level to test the relationship between the dependent variables “change in credibility” and “change in expected burden” and the independent variable “change in PTSD symptom severity.”

## Results

Three participating therapists missed the post-practical training assessment and two missed the 2-year follow-up assessment due to pregnancy leave or prolonged illness (unrelated to work as clinician).

### Influence of training on therapist-rated credibility, burden, and harm of trauma-focused treatment

**Fig. 1:**
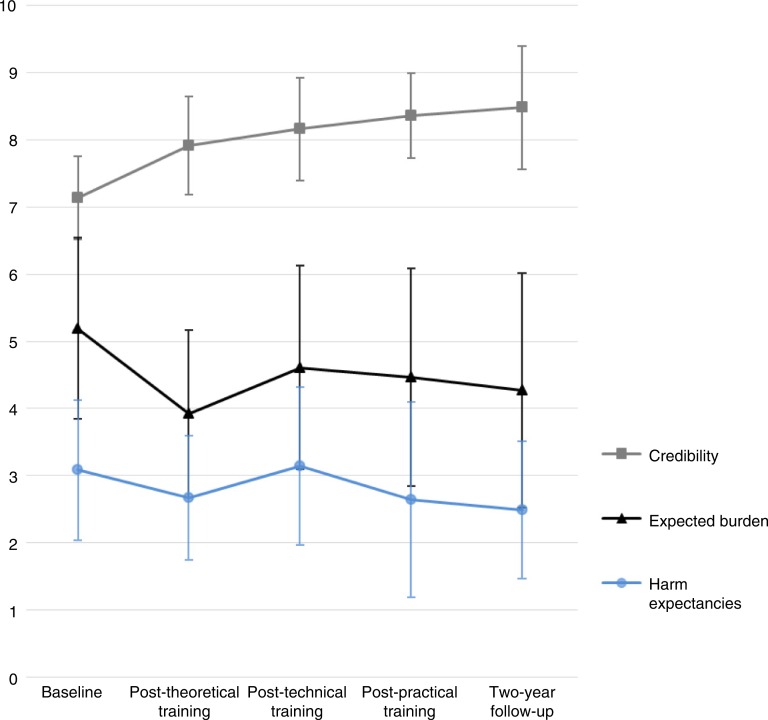
Estimated marginal means (SD) of therapist-rated credibility, expected burden, and harm expectancies of trauma-focused treatment at all time points (*N*=16). Range of mean scores is 0–10, with higher scores indicating higher therapist-rated credibility, greater expected burden, and more harm expectancies of trauma-focused treatment.

Figure 1 shows the estimated marginal means (produced by the mixed models analyses) for therapist-rated credibility, expected burden, and harm expectancies of trauma-focused treatment. Therapist-rated credibility of trauma-focused treatment increased significantly over time (*F*(4, 33.65)=11.75, *p<*0.001). Credibility increased significantly during the theoretical training (*M*_diff_=0.77, *t*(34)=3.36, *p*=0.002, 95% CI [0.30, 1.23]). This effect was sustained during the subsequent phases with small non-significant increases in credibility in every training phase. Therapist-rated burden expectancies showed a significant decrease over time (*F*(4, 31.35)=9.20, *p<*0.001). Burden expectancies decreased significantly after theoretical training (*M*_diff_=−1.27, *p*=0.004, 95% CI [−2.09, −0.43]). This was followed by a small, non-significant increase (*M*_diff_=0.69, *p*=0.088, 95% CI [−0.11, 1.48]) during technical training. Thereafter, the level of expected burden was relatively stable, showing no significant changes during the subsequent phases. Therapist-rated harm expectancies of trauma-focused treatment significantly decreased over time (*F*(4, 34.19)=4.44, *p*=0.005). After an initial small, non-significant decrease after theoretical training (*M*_diff_=−0.41, *p*=0.147, 95% CI [−0.96, 0.15]), harm expectancies showed a significant, but limited, increase during technical training (*M*_diff_=0.48, *p*=0.048, 95% CI [0.01, 0.93]) and then a limited (border) significant decrease during the practical training phase (*M*_diff_=−0.48, *p*=0.050, 95% CI [−0.97, −0.01]).

The 2-year follow-up survey (*n*=14) showed that 12 participating therapists (85.7%) still used trauma-focused treatments (PE or EMDR) in patients with psychotic disorders. In the last 2 years, the therapists had treated (on average) 12.3 (8.1) patients. The two participants that were no longer using trauma-focused treatments in psychosis had changed their job and were no longer working with this patient group. The majority of the participating therapists (78.6%) indicated that the supervised practical training had most strongly impacted their clinical behavior

### Influence of symptom severity on patient-specific credibility and burden of trauma-focused treatment

Table 1 shows that therapist-rated pre-treatment credibility and expected burden of trauma-focused treatment were negatively correlated. However, the pre-treatment symptom severity indicators showed little relationship with the pre-treatment ratings of credibility and expected burden. Only severity of PTSD symptoms was positively correlated with credibility, while no factor was associated with expected burden. In the linear mixed models analyses, none of the pre-treatment patient characteristics was significantly associated with the variability in either credibility or expected burden of trauma-focused treatment.

**Table 1:** Descriptive pre-treatment scores, Pearson product–moment coefficients, and linear mixed model results for the associations of patient characteristics with pre-treatment therapist-rated credibility and expected burden

	Pre-treatment	Post-treatment	Association with pre-treatment credibility	Association with pre-treatment burden
	Total *n*=79	Total *n*=65		
Credibility score, mean (SD)	7.4 (1.8)	7.5 (2.0)		
PPC			NA	−0.34**
Expected burden score, mean (SD)	3.9 (2.0)	3.7 (1.9)		
PPC			−0.34**	NA
Severity of PTSD (CAPS), mean (SD)	70.1 (15.8)			
PPC			0.22*	−0.04
LMM			*t*_73_=1.68, (0.098)	*t*_73_=−0.59, (0.552)
Severity of paranoid ideation (GPTS), mean (SD)	84.3 (32.5)			
PPC			0.11	0.12
LMM			*t*_73_=0.37, (0.709)	*t*_73_=1.04, (0.303)
Presence of AVH (AHRS), *n* (%)	33 (41.8)			
PPC			0.12	−0.16
LMM			*t*_73_=0.66, (0.513)	*t*_73_=−1.62, (0.110)
Presence of moderate-to-high suicide risk (MINI-plus), *n* (%)	36 (45.6)			
PPC			0.01	0.04
LMM			*t*_73_=−0.29, (0.773)	*t*_73_=0.29, (0.768)
Level of social functioning (PSP), mean (SD)	52.5 (12.1)			
PPC			0.02	−0.18
LMM			*t*_73_=0.46, (0.644)	*t*_73_=−1.19, (0.235)

** Correlation is significant at the 0.01 level (two-tailed)

* correlation is significant at the 0.05 level (two-tailed). AHRS, Auditory Hallucination Rating Scale; AVH, Auditory Verbal Hallucinations; CAPS, Clinician Administered PTSD Scale; GPTS, Green et al. Paranoid Thought Scales; LMM, Linear Mixed Models analyses; MINI-plus, Mini-International Neuropsychiatric Interview-Plus; PPC, Pearson product–moment coefficient; PSP, Personal and Social Performance Scale; PTSD, posttraumatic stress disorder; SD, standard deviation.

### Influence of treatment outcome on patient-specific credibility and burden of trauma-focused treatment

Of the 79 patients treated by the 16 therapists, 8 (10.1%) did not attend the post-treatment assessment of severity of PTSD symptoms. Also, 14 patients (17.7%) had no post-treatment therapist ratings of credibility and expected burden, either because the patient dropped out of treatment or because the therapist became ill. For 63 patients (79.7%), all data were present. Although the mean CAPS severity score for these patients showed a significant change during treatment (mean change −34.7, *t*[62]=12.83, *p<*0.001), the results of the paired-samples *t*-tests showed no significant change in either therapist-rated credibility (*t*[62]=−0.16, *p*=0.880) or burden expectancies (*t*[62]=0.59, *p*=0.550) during treatment (see Table 1 for mean scores). Moreover, the magnitude of the change in PTSD symptom severity during treatment (i.e., treatment outcome) was not significantly correlated with the change in therapist-rated credibility (*r*=0.04, *n*=63, *p*=0.739) or burden (*r*=−0.12, *n*=63, *p*=0.347). The mixed model analyses with a correction for therapist level also showed results far from significance.

All the analyses in this section were repeated with the last observation carried forward (no change, *n*=79) as sensitivity analyses, and all yielded results similar to the original analyses.

## Discussion

To our knowledge, this is one of the first studies to examine the differential impact of theoretical, technical, and supervised practical training on therapists’ general beliefs concerning trauma-focused treatments and also the first study to test the influence of symptom severity and treatment outcome on these beliefs. The results of this feasibility study show that specialized trauma-focused treatment training with a subsequent trajectory of technical and supervised practical training resulted in a significant increase in therapist-rated credibility and a decrease in the expected burden and harm expectancies of trauma-focused treatment. These effects were sustained up to 2-year follow-up, and all the therapists that were still working with patients with psychosis were still using trauma-focused treatments with these patients. During the supervised practical training phase, therapists’ patient-specific beliefs concerning credibility and expected burden were not affected by the severity of symptoms. Patient-specific credibility and expected burden of trauma-focused treatment did not change during treatment, regardless of the treatment outcome. Therefore, these findings lend support for the notion that specialized training has a long-term positive effect on therapists’ beliefs concerning trauma-focused treatment.

Therapists’ credibility of trauma-focused treatment showed an increasing trend over the course of training, with a significant increase during theoretical training. Ceiling effects may have influenced the slope, as the mean credibility score after theoretical training was relatively high. These effects are in line with a recent naturalistic study that found both training (large effect) and post-training telephone consultation (moderate effect) to increase credibility of PE (Ruzek et al., 2016). This study also reported associations between therapist-rated credibility of PE and actual usage of it (Ruzek et al., 2015), demonstrating the importance of this factor. These results underline the necessity of providing therapists with empirical information about the effects and rationale of trauma-focused treatments, and of familiarizing them with the basic procedures, techniques, and skills (Karlin et al., 2010).

There was a clear reduction in therapists’ expected burden of trauma-focused treatment during theoretical training and a partial (non-significant) rebound during the technical training when therapists started to treat patients. This partial recovery of burden expectancies and the fact that these showed no further significant decrease during the practical training phase or follow-up may be explained by the fact that a certain level of burden of trauma-focused treatment is probably realistic—especially when considering that the therapists (although experienced) were novices to trauma-focused treatment and immediately started treating a patient group characterized by severe PTSD, many comorbidities, and severe childhood traumas (Van den Berg et al., 2015). These findings are at odds with the study by Ruzek et al. (2016), in which burden beliefs significantly decreased during a 6- to 9-month telephone consultation phase, but not during theoretical training.

Interestingly, there was an increase in harm expectancies during the technical training phase and a decrease during the supervised practical training phase. Ruzek et al. (2016) found moderate reductions in harm expectancies during both (theoretical) training and post-training telephone consultation. It is difficult to compare these results, since their post-training consultation comprised both our technical and practical training phase. Moreover, our results are likely to have been influenced by floor effects, since the mean baseline score of therapists’ harm expectancies was rather low. This may be a specific sample characteristic since all the therapists in this study were experienced in working with complex and severe patients, and all agreed to participate in a trial for trauma-focused treatments in psychosis. This may have resulted in a sample of therapists that were less anxious than “average” therapists. Greater anxiety sensitivity has been associated with a tendency to exclude patients from exposure therapy (Meyer et al., 2014).

During the supervised practical training (within the context of a trial), the characteristics of specific patients (symptom severity and level of social functioning) and treatment outcome had no influence on therapists’ credibility and burden beliefs concerning trauma-focused treatment. The only significant association was a positive correlation between pre-treatment PTSD symptom severity and pre-treatment credibility of trauma-focused treatment which, in the multiple regression analysis, was lost after correction for the variability explained by the other pre-treatment patient characteristics. This positive correlation tentatively suggests that therapists, with high mean scores of credibility of trauma-focused treatment at that time, may have reasoned that with severe PTSD trauma-focused treatment was probably going to be effective. Greater severity of pre-treatment PTSD was indeed found to be related to a greater reduction in PTSD symptoms during treatment in participants with severe mental illness (Mueser et al., 2008) and in several general PTSD samples (Elliott, Biddle, Hawthorne, Forbes, & Creamer, 2005; Foa, Riggs, Massie, & Yarczower, 1995; Forbes, Creamer, Hawthorne, Allen, & McHugh, 2003; Karatzias et al., 2007; Rizvi, Vogt, & Resick, 2009; Thrasher, Power, Morant, Marks, & Dalgleish, 2010), but not in others (De Kleine, Hendriks, Smits, Broekman, & Van Minnen, 2014; Speckens, Ehlers, Hackmann, & Clark, 2006). The fact that, in this complex patient group, therapists were not influenced by specific patient characteristics gives cause for optimism; this indicates that extensive specialized training may have durable effects that are independent of specific sample characteristics.

The present results suggest that different elements of training may have a differential impact on therapists’ beliefs. Interestingly, at 2-year follow-up, most of the therapists indicated that the supervised practical training was the most important factor in shaping their clinical behavior. This is in accordance with the recommendations of Karlin et al. (2010), who also stressed the importance of ongoing consultation after training. It is possible that trauma-focused therapists are not so different from their patients; similar to their patients, they may benefit from a “coach” who knows the process, provides information that increases their credibility of the treatment, and relativizes burden expectancies based on research findings and extensive clinical experience. In other words, a guide who stimulates them to test new behaviors and falsify their harm expectancies (Craske, Treanor, Conway, Zbozinek, & Vervliet, 2014; Rief et al., 2015). Future training programs aimed at disseminating trauma-focused treatments would benefit from adopting these cognitive behavioral principles and practices, to actively expose therapists to using trauma-focused treatments with “difficult” patients, and to stimulate therapists to investigate and challenge their negative harm expectancies of trauma-focused treatment in patient groups with severe and complex symptoms (Farrell et al., 2013). In doing this, it is important to realize that although trauma-focused treatment is safe, perceiving a certain level of burden is probably realistic.

This feasibility study has several limitations. The most important limitations (related to the fact that the data were collected as part of a RCT) are the fact that participating therapists were not randomly selected, the lack of a control group, and the small sample size. A strength of this contextual factor is that fidelity to the protocol was high; however, this also limits the generalizability. Moreover, participating therapists voluntarily participated in a trial on trauma-focused treatment in psychosis, despite (at that time) limited empirical evidence regarding its efficacy and safety. This may have resulted in ceiling and floor effects. Furthermore, participating therapists concurrently received PE and EMDR training, which might have had a differential influence on their beliefs. The data for these two trauma-focused treatments were pooled as we could not isolate carry-over effects. Unfortunately, at the patient level we did not assess harm expectancies. Also, patient-specific burden expectancies (rated by the therapists) only concerned therapist-rated burden to the therapist and not to the patient. Future studies could include these latter assessments. Finally, we used non-validated measures to assess therapist's beliefs, although internal consistency scores were satisfactory.

In conclusion, the present findings support the notion that specialized trauma-focused treatment training, including acquisition of experience, increases credibility and reduces beliefs about burden and harm. This is underlined by the finding that the effects were sustained on the long term and were unaffected by specific patient characteristics and treatment outcome. Future studies could use a similar design with larger samples of frontline therapists in clinical practice. These studies should include a control group and may test whether the level of experience influences training effects. These studies could also compare theoretical and technical training with training that is augmented with an expert supervision trajectory. It is important to establish what beliefs are most strongly related to long-term clinical behavior and what elements of training have the strongest influence on these beliefs (Ruzek et al., 2015). With regard to dissemination efforts, future studies could also examine whether less intensive training programs produce similar results.
